# Structure of a new glycyrrhiza polysaccharide and its immunomodulatory activity

**DOI:** 10.3389/fimmu.2022.1007186

**Published:** 2022-09-27

**Authors:** Yu Wu, Hui Zhou, Kunhua Wei, Tao Zhang, Yanyun Che, Audrey D. Nguyễn, Sakshi Pandita, Xin Wan, Xuejie Cui, Bingxue Zhou, Caiyue Li, Ping Hao, Hongjun Lei, Lin Wang, Xiaonan Yang, Ying Liang, Jiaguo Liu, Yi Wu

**Affiliations:** ^1^ Ministry of Education (MOE) Joint International Research Laboratory of Animal Health and Food Safety, College of Veterinary Medicine, Nanjing Agricultural University, Nanjing, China; ^2^ Institute of Traditional Chinese Veterinary Medicine, College of Veterinary Medicine, Nanjing Agricultural University, Nanjing, China; ^3^ Guangxi Key Laboratory of Medicinal Resources Protection and Genetic Improvement/Guangxi Engineering Research Center of Traditional Chinese Medicine (TCM) Resource Intelligent Creation, Guangxi Botanical Garden of Medicinal Plant, Nan Ning, China; ^4^ Beijing Key Laboratory of Traditional Chinese Veterinary Medicine, Beijing University of Agriculture, Beijing, China; ^5^ Engineering Laboratory for National Healthcare Theories and Products of Yunnan Province, College of Pharmaceutical Science, Yunnan University of Chinese Medicine, Kunming, China; ^6^ Department of Biochemistry and Molecular Medicine, Davis Medical Center, University of California, Davis Medical, Sacramento, CA, United States; ^7^ Animal Science and Veterinary College, Jiangsu Vocational College of Agricultural and Forestry, Zhenjiang, China

**Keywords:** glycyrrhiza polysaccharide, structural characterization, immunomodulatory activity, dendritic cells, toll-like receptor

## Abstract

A component of licorice polysaccharide (GPS-1) was extracted from licorice, its primary structure was identified and characterized for the first time, and its immunomodulatory activity was studied. Crude licorice polysaccharide was isolated and purified by DEAE sepharose FF ion-exchange column chromatography and Chromdex 200 PG gel filtration column chromatography to obtain a purified Glycyrrhiza polysaccharide named GPS-1. NMR and methylation analysis revealed that GPS-1 is composed of homogalacturonan (HG)-type pectin with 4)-D-GalpA-(1 as the backbone. This study of GPS-1 also examined its significant role in regulating immune activity *in vitro* and *in vivo*. As a result, GPS-1 promoted the secretion of IFN-γ and IL-4 in mice and increased the proportion of CD3^+^CD4^+^ and CD3^+^CD8^+^ T lymphocytes in their spleens. Dendritic cells (DCs) treated with GPS-1 showed promotion of DC maturation, antigen presentation, and phagocytic capacity. The results suggest that GPS-1 is a potential immunomodulator that stimulates the immune system by regulating multiple signaling pathways. Combined with our characterization of the primary structure of GPS-1, the present investigation provides the basis for future study of the form-function relationship of polysaccharides.

## 1 Introduction

Licorice is not only a widely used food but also traditional Chinese medicine (1). The use of licorice has been recorded in Europe as early as 2100 BC and is now a well-known natural food sweetener (2). Licorice’s medicinal benefits are now recorded in the pharmacopeias of China, South Korea, and Japan (3). Studies of active ingredients in licorice have found that polysaccharides are important components (4, 5) and that one of the main functions of licorice polysaccharides is to improve immunity (4, 6, 7). Another investigation found that licorice polysaccharides could dose-dependently counteract cyclophosphamide-induced immunosuppression in mice (8). Researchers have also demonstrated a positive correlation between the immune activity of polysaccharides and their uronic acid content (9). Our previous study also confirmed that glycyrrhiza polysaccharides can prolong the efficacy and duration of the Newcastle disease virus (NDV) vaccine (10) and improve the intestinal immune function and microbial composition of roosters (4). In addition, the glycyrrhiza polysaccharide (GiP-B1) can cause significant phenotypic changes in dendritic cells (DCs) and the improvement of bioactive functions with a mechanism of action related to the TLRs/NF-B signaling pathway (11). In the present study, the purified glycan GPS-1 was isolated from licorice (*Glycyrrhiza uralensis*), then its structure was identified using methylation and NMR analysis. The immunomodulatory mechanisms and pathways of GPS-1 were also explored *in vivo* with mice and *in vitro* with dendritic cells.

## 2 Materials and methods

### 2.1 Separation and purification of glycyrrhiza polysaccharides

**Figure 1:**
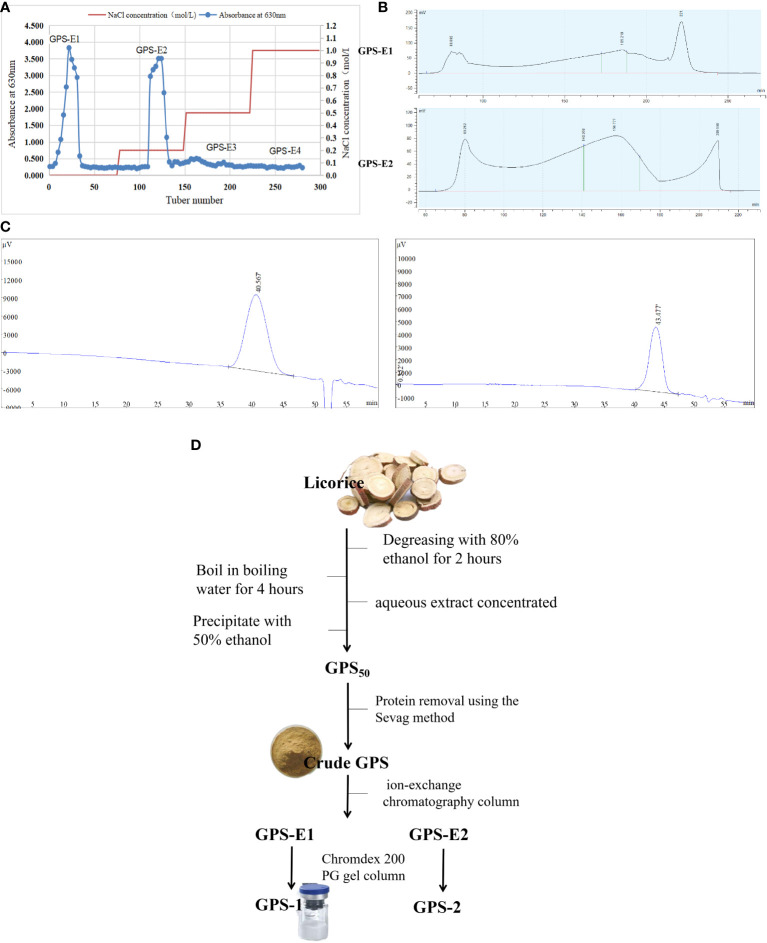
**(A)** Elution profile of GPS50 ion-exchange column chromatography. **(B)** Elution profiles of samples GPS-E1 and GPS-E2 gel filtration column chromatography **(C)** The HPGPC chromatogram of the GPS-1 (left) and GPS-2 (right). **(D)** The specific flow chart of GPS-1 extraction.

Using our previously reported methods (10), crude Glycyrrhiza polysaccharide (GPS) was extracted by boiling water from licorice (*Glycyrrhiza uralensis*) and then precipitated with 50% ethanol into GPS_50_ extract (12). Proteins were removed five times, sequentially, using the Sevag method. The final freeze-dried samples were stored at -20°C. Next, 30 g of decorin GPS_50_ was weighed, dissolved with 30 mL distilled water (dH_2_O), and centrifuged at 8000 rpm for 10 min. Then, the precipitate was removed and filtered with a 0.45 µm microporous membrane. The filtrate was then run through a balanced ion-exchange chromatography column and eluted according to different salt concentrations (0.0 M NaCl, 0.2 M NaCl, 0.5 M NaCl, and 1.0 M NaCl) at a speed of 5.0 mL/min. At the same time, the eluents were collected (10 mL/tube) for 100 tubes, and then analyzed using the reported method (12). The eluents from the same peak (1-50 tubes and 51-100 tubes) were collected, combined, and concentrated, respectively, and then lyophilized to provide two samples (GPS-E1 and GPS-E2). The two samples were subjected to Chromdex 200 PG gel column and eluted with ultrapure water at a flow rate of 2mL/min. Each eluent was collected (10 mL/tube) and detected by the same method (12). The relevant eluents (170-190 min and 140-170 min) were gathered, combined, concentrated, and freeze-dried in a vacuum to give purified polysaccharide GPS-1 and GPS-2 (Refer to **Figure 1D**).

### 2.2 Characterization of the GPS-1 study

Due to the low sample yield of GPS-2 after purification, GPS-1 was studied in subsequent tests.

#### 2.2.1 Molecular weight of GPS-1

Purified GPS-1 polysaccharide was mixed with 0.05 M NaCl to prepare a 5 mg/mL test solution, then filtered using a 0.22 µm membrane. Tests of the molecular weight of GPS-1 were performed using high-performance gel permeation chromatography (HPGPC, Agilent 1100 series HPLC, Agilent Technologies, Santa Clara, CA, USA). The fractions were isolated by OHpak SB-803 HQ column at a flow speed of 1 mL/min and detected by an Agilent G1362A Refractive Index Detector at room temperature. The purchased dextran standards with a series of molecular weights (1000, 5000, 12000, 25000, 50000, 80000, 150000, 270000, 410000, 670000 Da, Sigma-Aldrich, Merck, Germany) were used to plot the calibration curve. The molecular weight of GPS-1 was calculated according to the standard linear regression equation.

#### 2.2.2 Monosaccharide composition of GPS-1

The monosaccharide composition of GPS-1 was analyzed by the reported method in the reference with a few modifications (13). The GPS-1 polysaccharide sample (5 ± 0.05 mg) was weighed, added into a reaction flask, and hydrolyzed with trifluoroacetic acid (TFA, 2mL of 1M) at 121 °C for 4h. After removing the remaining acid by flushing with N_2_, the reaction products were evaporated to dryness under reduced pressure. The residue was dissolved by methanol (2 mL) and dried by flushing N_2_ three times to remove the TFA completely, and then re-dissolved in fresh distilled water (1 mL). The solution (5 µL) was analyzed by high-performance anion-exchange chromatography (HPAEC) on a CarboPac PA-20 anion-exchange column (3 by 150 mm; Dionex) using a pulsed amperometric detector (PAD; Dionex ICS 5000 system). The isolation conditions are as follows: flow rate, 0.5 mL/min; injection volume, 5μL; solvent system, B: (0.1M NaOH, 0.2M NaAc); gradient program, 95:5 V/V at 0 min, 80:20 V/V at 30 min, 60:40 V/V at 30.1 min, 60:40 V/V at 45min, 95:5 V/V at 45.1 min, 95:5 V/V at 60 min. Data were acquired on the ICS5000 (Thermo Scientific) and processed using chameleon 7.2 CDS (Thermo Scientific). Quantified data were output into excel format.

And the monosaccharide composition of GPS-1 was analyzed according to the literature-reported method (13). Chromatographic data were processed using Chromeleon™ chromatography data system software. In the standard sample ion chromatogram and the sample ion chromatogram, the abscissa is the detection retention time (Time, in minutes), and the ordinate is the response value of the ion detection (Response, in nC).

The content of each component in the sample (µg/mg) = C * V * F/M, where C is the instrument read concentration in µg/mL, V is the sample extract volume in mL, F is the dilution factor, and M is the total sample volume in mg.

#### 2.2.3 Methylation analysis of GPS-1

Next, 10 mg of dried GPS-1 and 20 mg of dried NaOH were dissolved in 2000 µL DMSO to give a mixture. The mixture was kept at 25 °C for 10 h, and then reacted with an equal volume of methyl iodide for 3 h. This methylation reaction was repeated for five times. After the reaction, the mixture was extracted with an equal volume of CHCl_3_ five times, and the CHCl_3_ extract solvent was combined and evaporated to dryness to provide a residue. The residue was hydrolyzed with 1 mL of TFA (2 M) at 120 °C for 2 h and then evaporated under vacuum to dryness. The sample was treated with NaBH_4_ (60 mg) for 8h at room temperature and then neutralized with acetic acid (0.1 M). Afterward, the residue was dried at 101 °C and the product was dissolved in CHCl_3_ and detected by an Agilent 7890A-5977B GC-MS instrument (Agilent Technologies Inc. CA, USA) equipped with an RXI-5 SIL MS column (30 m × 0.25 mm × 0.25 μm). The oven temperature was maintained at 140 °C for 2.0 min at first, and then increased from 140 °C to 230 °C at a speed of 3 °C/min, then maintained at 230 °C for 3 min. The carrier gas was Helium with a purity of 99.999%, and the flow rate was 1.0 mL/min.

#### 2.2.4 Nuclear magnetic resonance (NMR) analysis of GPS-1

The lyophilized sample (30 mg) was dissolved in 0.5 mL of D_2_O standard. A Bruker Ascend 600 spectrometer (Bruker, Stockholm, Sweden) at 600 MHz was used to determine the one-dimensional nuclear magnetic (^1^H-NMR, ^13^C-NMR, and ^13^C-DEPT-135) and two-dimensional NMR [^1^H-^1^H COSY (correlated spectroscopy), TOCSY (total correlation spectroscopy), HSQC (heteronuclear single quantum coherence), HMBC (heteronuclear multiple bond correlation), and NOESY (nuclear Overhauser effect spectroscopy)] of GPS-1. Tetramethylsilane was used as an internal standard.

### 2.3 *In vivo* immunomodulatory activity study of GPS-1

#### 2.3.1 Grouping and treatment of mice

A total of 20 male ICR mice were purchased from the Comparative Medicine Center of Yangzhou University (License Number: SCXK (Su) 2017-007) at 5 weeks of age. They were randomly divided into 4 groups, each with 5 mice. The control group was given only a regular diet, while the treatment groups received a regular diet plus oral GPS-1 at a low (GPS-L), medium (GPS-M), or high-dose (GPS-H). These groups received 300, 450, and 600 mg/kg/d, respectively, for 14 consecutive days. All mice were housed at the Animal Care Center of Nanjing Agricultural University under the guidelines of the Institutional Animal Care and Use Committee Institutional Animal Care and Use Committee (IACUC), as detailed in the IACUC-approved protocol (No.: 2020BAD25D34).

#### 2.3.2 Determination of the cytokines in the serum

On day 14, all 5 mice in each group were sacrificed, and blood serum was sampled. The IFN-γ and IL-10 averages in each group were determined according to ELISA kit instructions (Wuhan Feen Biotechnology Co., LTD, product code: EM0093;EM0100).

#### 2.3.3 Histological analysis

Each of these 5 mice per group also had their thymus, spleen, and small intestines collected on day 14 and preserved in 4% formaldehyde (Beijing Solarbio Biological Technology Co., LTD). These tissues were fixed, dewaxed, stained, mounted and mounted for H&E histological analysis.

#### 2.3.4 Mouse spleen lymphocyte typing

After spleens were collected on day 14 (n = 5 per group), primary spleen lymphocytes were extracted according to literature reports (4, 10). The stained cells were incubated with lipopolysaccharides (LPS, from Sigma) and CD3-FITC, CD4-PC, and CD8-APC (eBioscience™, AB_2536505) for 24 h, then measured using a flow cytometer (BD Biosciences AccuriC6, USA).

### 2.4*In vitro* modulation of DCs immune activity by GPS-1

#### 2.4.1 Preparation of mouse bone marrow-derived dendritic cells

Male Balb/c mice were purchased from the Comparative Medicine Center of Yangzhou University (License No.: SCXK (Su) 2017-007) at 5 weeks of age. The tibia and femur of each hindlimb were removed and the bone marrow cavity was flushed using sterile PBS to obtain bone marrow multifunctional stem cell irrigation fluid. The precursor dendritic cells (DCs) were isolated according to the relevant methods reported in prior literature (14, 15). First, the irrigation fluid was centrifuged (1200rpm, 5min) to obtain cell pellets, and after re-suspension,bone marrow multifunctional stem cell were obtained by using mouse monocyte separation solution (Beijing Solarbio Biological Technology Co., LTD).These cells were then cultured in DMEM media (Gibco,USA) with 1% penicillin/streptomycin (Gibco,USA), 10% FBS (Gibco,USA), 1% Mouse-derived IL-4 stimulating factor (Gibco,USA,Cat#214-14),and 1% mouse-derived Colony-stimulating factor (acting on macrophages) GM-CSF stimulating factor (Gibco,USA, Cat#815-03) for 7 days at 37 °C and 5% CO_2_ in a cell incubator. Immature DCs can be obtained after 7 days of induced differentiation and culture.

#### 2.4.2 Determination of the safe concentration of GPS-1

Immature DCs were grown in 96-well plates to a confluence of 4.8 × 10^5^ cells per mL and treated with GPS-1 at different concentrations (2000, 1000, 500, 250, 125, 62.5, 31.25, 15.625, and 7.814 µg/mL), plus a blank control containing DCs in complete media. N = 6 wells per group, incubated at 37 °C and 5% CO_2_ in a cell incubator for 36 hours. Absorption values were determined using the MTT method. Cell survival was calculated using the equation below, where OD is the optical density:

Cell viability (%) = OD in the drug group/OD of 100% in the blank group.

#### 2.4.3 Determination of cytokines in dendritic cells

GPS-1 doses of 500, 250, and 125 µg/mL were added to 1 × 10³ DCs per well in 96-well plates and incubated for 36 hours, along with a DC negative control group in complete media, and positive control of DCs treated with LPS. N = 4 for all groups. Cell culture supernatants were collected, and their IFN-γ, TNF-α, IL-1β, and IL-10 (Wuhan Feen Biotechnology Co., LTD, product code: EM0093; EM0183; EM0109; EM0100)contents were determined by ELISA.

#### 2.4.4 Determination of the phagocytic capacity of dendritic cells

Dendritic cells were incubated in 96-well plates at 37°C and 5% CO_2_ for 8 hours with either GPS-1 (500, 250, or 125 µg/mL), or complete media (Control), with n =4 for all groups. 20 µL of florescent-labeled ovalbumin antigen (OVA-FITC) was added to each well to mimic natural antigen phagocytosis by DCs. After 8 hours of incubation, cells were centrifuged at 1000 rpm/min for 10 min. Excess antigen was washed off with 1 ml PBS before cells were sorted by flow cytometry to determine the proportion of viable DCs. The optimal concentration of GPS-1 to stimulate DC antigen phagocytosis was determined to be 500 µg/mL.

Cells from the 500 µg/mL GPS-1 and blank (Control) groups were added to confocal dishes. The same OVA-FITC treatment described above was used to simulate antigen phagocytosis again. Nuclei were labeled with 5 µL of DAPI dye solution and incubated at 4°C for 10 min. Afterward, excess DAPI dye solution was rinsed twice with 1 ml PBS. Finally, a confocal laser microscope was used to compare the changes in antigen intake between the 500 µg/mL GPS-1 group and the control.

#### 2.4.5 Determining the presentation ability of the dendritic cells

24-well plates were used to incubate 1 × 10^4^ DCs/mL, 2 ml of cells in complete media per well (n = 4). Different concentrations of GPS-1 were added to achieve final concentrations of 500, 250, and 125 µg/mL, except for a blank (Control) group without GPS-1. All groups were incubated for 36 hours at 37 °C and 5% CO_2_. 15 × 10^4^ DCs per well were seeded in a 6-well plate and treated with 3 µl per well of 3 mouse antibodies (1 µl of each): anti-CD11c-PE-Cyanine5, anti-CD86-PE, and anti-CD80-FITC were incubated at 4°C for 30 min. The excess antibodies were subsequently washed off the DCs twice with 1 ml PBS. Then, flow cytometry was used to determine the expression levels of CD80 and CD86 costimulators on the surface of DCs from each group.

### 2.5 Dendritic cell active pathway mechanisms targeted by GPS-1

DCs were treated with 500 µg/mL of GPS-1 for 36 hours alongside a negative control group without GPS-1. DCs’ total RNA was extracted by TRizol, followed by RNA quality detection using a NanoDrop spectrophotometer and an Agilent 2100 bioanalyzer. Fragments Count for each gene in each sample was counted by HTSeq (v 0.5.4 p3) software, and FPKM (Fragments Per Kilobase Per Million Mapped Fragments) was then calculated to estimate the expression level of genes in each sample. For analysis of DEGs, DESeq/DESeq2 was employed to detect the differential expression between the two groups using a model based on the negative binomial distribution. The P value was assigned to each gene and adjusted as q value by the Benjamini and Hochberg method for controlling the false discovery rate (FDR). Genes with q < 0.05 were defined as DEGs.The GO (Gene Ontology, http://geneontology.org) enrichment of DEGs was conducted by the hypergeometric test, in which P value was calculated and adjusted as q value. GO terms with q < 0.05 were considered to be significantly enriched. For pathway enrichment analysis, all DEGs were assigned to terms in KEGG (Kyoto Encyclopedia of Genes and Genomes, http://www.kegg.jp) database and searched for significantly enriched KEGG terms with the same analytic approach.

### 2.6 Statistical analysis

All results are expressed as the mean ± standard deviation, graphed using GraphPad Prism 6.0 software and SPSS 18.0 software for ANOVA multiple comparisons. P< 0.05 is considered statistically significant in this analysis.

## 3 Results

### 3.1 Separation and purification of licorice polysaccharide

The elution curves displayed in **Figure 1** were obtained by gradient elution of GPS_50_ through an ion-exchange chromatography column. The order of samples collected was: GPS-E1 (deionized water elution), GPS-E2 (0.2M NaCl elution), GPS-E3 (0.5M NaCl elution), and GPS-E4 (1.0M NaCl elution). The total sugar content of each was then determined by the anthrone-sulfuric acid method. As shown in **Figure 1A**, the peaks eluted with deionized water and 0.2M NaCl solution from the Ion-exchange column were selected for purification by Chromdex 200 PG gel column chromatography. As depicted in **Figure 1B**, the eluates of GPS-E1 between 210 and 230 min, and those of GPS-E2 between 140-170 min were collected and named GPS-2 and GPS-1, respectively. As the result, the total yield of GPS-1 and GPS-2 were 0.78% and 0.07%, and their sugar content was 98.95% and 97.03%, respectively.

Two single symmetrical and narrow peaks were exhibited in the HPGPC spectrum in **Figure 1C**, which indicated that GPS-1 and GPS-2 were homogeneous. The weight-average molecular weight (M_w_) of GPS-1 and GPS-2 were 26.4 kDa and 8.9 kDa, and the number average molecular weight (M_n_) of GPS-1 and GPS-2 were 18.0 kDa and 6.4 kDa, respectively. Additionally, the M_w_/M_n_ value of GPS-1 and GPS-2 were 1.47 and 1.35, respectively. However, because of the poor weight of GPS-2 (less than 5 mg), further investigation was processed only on the structure and biological activities of GPS-1.

Comparison of peak heights and the presence or absence of peaks between the standard monosaccharide chromatogram and the monosaccharide chromatogram of the GPS-1 sample (**Figure 1C** led to the conclusion that GPS-1 is composed of 74.39% galacturonic acid, 10.12% arabinose, 6.36% rhamnose, 4.85% galactose, 1.52% mannuronic acid, 1.34% glucose, 0.92%, fucose, and 0.51% xylose. Roughly two-thirds of the monomers that make up GPS-1 are galacturonic acid. Arabinose, the second most prevalent, accounts for only 10.12%. See **Table 1** for a complete summary of GPS-1 monosaccharides, including the concentrations calculated using peak areas.

**Table 1:** Monosaccharide composition and ratio of GPS-1.

Monosaccharide	Mass conversion result (ug/mg)	The percentage of each component (%)
Fuc	2.84	0.92
Ara	31.20	10.12
Rha	19.59	6.36
Gal	14.94	4.85
Glc	4.12	1.34
Xyl	1.56	0.51
Man	None	None
Fru	None	None
Rib	None	None
Gal-UA	229.23	74.39
Gul-UA	None	None
Glc-UA	4.68	1.52
Man-UA	None	None

### 3.2 Structure of GPS-1

#### 3.2.1 GPS-1 methylation analysis

According to the results of complete acid hydrolysis methylation analysis, the sequence of sugar residues in GPS-1 is mainly 4-Gal(p)-UA, and the multiple ends are t-Rha(p), t-Ara(f), t -Fuc(p), t-Xyl(p), t-Gal(p), and t-Gal(p)-UA. The analysis indicates that GPS-1 is composed of multiple branched chains, with a complex secondary structure illustrated in **Table 2**.

**Table 2:** The sugar residue connection mode of GPS-1.

Connection method	Derivative name	molecular weight (MW)	relative molar ratio (%)
t-Rha(p)	1,5-di-O-acetyl-6-deoxy-2,3,4-tri-O-methyl rhamnitol	293	2.336
t-Ara(f)	1,4-di-O-acetyl-2,3,5-tri-O-methyl arabinitol	279	4.328
t-Fuc(p)	1,5-di-O-acetyl-6-deoxy-2,3,4-tri-O-methyl fucitol	293	1.174
t-Xyl(p)	1,5-di-O-acetyl-2,3,4-tri-O-methyl xylitol	279	0.944
2-Rha(p)	1,2,5-tri-O-acetyl-6-deoxy-3,4-di-O-methyl rhamnitol	321	1.246
3-Rha(p)	1,3,5-tri-O-acetyl-6-deoxy-2,4-di-O-methyl rhamnitol	321	1.123
t-Gal(p)	1,5-di-O-acetyl-2,3,4,6-tetra-O-methyl galactitol	323	4.051
t-Gal(p)-UA	1,5-di-O-acetyl-2,3,4,6-tetra-O-methyl galactitol	323	6.076
5-Ara(f)	1,4,5-tri-O-acetyl-2,3-di-O-methyl arabinitol	307	2.797
4-Ara(p)	1,4,5-tri-O-acetyl-2,3-di-O-methyl Araitol	307	1.352
4-Gal(p)-UA	1,4,5-tri-O-acetyl-2,3,6-tri-O-methyl galactitol	351	61.675
4-Glc(p)	1,4,5-tri-O-acetyl-2,3,6-tri-O-methyl glucitol	351	1.942
4-Glc(p)-UA	1,4,5-tri-O-acetyl-2,3,6-tri-O-methyl glucitol	351	1.942
6-Gal(p)-UA	1,5,6-tri-O-acetyl-2,3,4-tri-O-methyl galactitol	351	4.955
3,4-Glc(p)	1,3,4,5-tetra-O-acetyl-2,6-di-O-methyl glucitol	379	0.679
3,4-Glc(p)-UA	1,3,4,5-tetra-O-acetyl-2,6-di-O-methyl glucitol	379	0.679
2,4-Gal(p)-UA	1,2,4,5-tetra-O-acetyl-3,6-di-O-methyl galactitol	379	1.335
3,6-Gal(p)	1,3,5,6-tetra-O-acetyl-2,4-di-O-methyl galactitol	379	1.367

#### 3.2.2 GPS-1 one-dimensional NMR analysis

**Figure 2:**
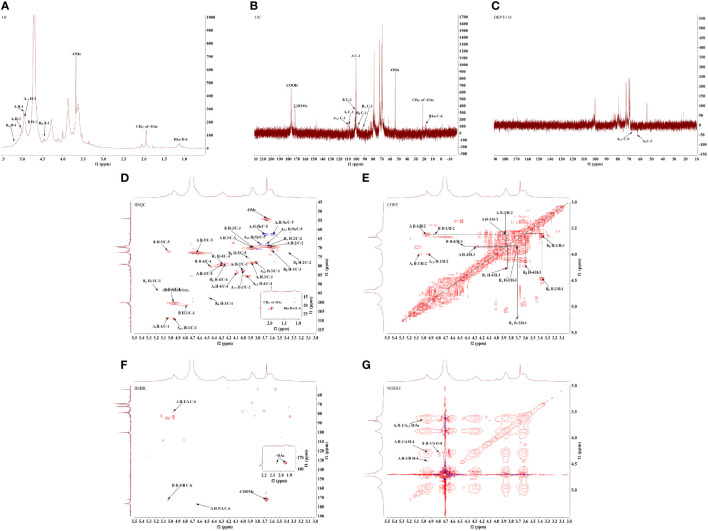
GPS-1 one-dimensional nuclear magnetic resonance spectrum **(A)** 1H-NMR. **(B)** 13C-NMR. **(C)**13C-DEPT.GPS-1 2D NMR spectrum **(D)** HSQC. **(E)** 1H-1H COSY. **(F)** HMBC. **(G)** NOESY.

One-dimensional hydrogen nuclear magnetic resonance spectroscopy (^1^H-NMR) (**Figure 2A**), and carbon spectroscopy (^13^C-NMR) (**Figure 2B**), were used to further identify the glycosidic bond configuration of GPS-1 samples. In the ^1^H-NMR spectrum (the peals at 5.18, 5.04, 4.96, 4.95, 4.80, and 4.47 ppm were assigned to H-1 of R_α_, A_t_, A, A_1, 5_, B and R_β_ residues. Peaks between 3.36 and 5.02 ppm belong to sugar ring protons. In the ^13^C DEPT 135 spectrum (**Figure 2C**, negative peaks at 62.43 ppm were assigned to the A_t_-C5. The main anomeric carbon peaks in the ^13^C NMR spectrum observed at 108.90, 108.47, 101.55, 100.43, 97.65 and 93.69 ppm were corresponding to C-1 of A_1, 5_, A_t_, B, A, R_β,_ and R_α_ sugar residues, signals at 176.94, 176.49 (× 2) and 172.20 ppm were attributed to the carbonyls of A, R_β_/R_α_ and B galacturonic residues, peaks at 75.69 (× 2), 72.83, 72.12, 68.17 and 62.68 ppm belonged to the C-5 of R_β_/R_α_, A, B, A_1, 5_ and A_t_, while other signals between 60 and 90 ppm were attributed to C-2, C-3 and C-4 of the sugar rings. These data confirmed the existence of Arabinosyl and galacturonic residues of GPS-1 (16).

#### 3.2.3 GPS-1 2D NMR analysis

According to the monosaccharide composition and methylation analysis results of the polysaccharide samples, the HSQC (**Figure 2D** and 1H-1H COSY (**Figure 2E**) NMR spectra showed that there were multiple signal peaks in the anomeric region, which were significant and could be used for structural analysis. The chemical shifts of the signals in the anomeric region were respectively δ 4.96/100.43 ppm, δ 4.80/101.55 ppm, δ 4.95/108.90 ppm, δ 5.04/108.47 ppm, δ 5.18/93.69 ppm, δ 4.47/97.65 ppm, named as sugar residues A, B, A1,5, At, and the reducing end groups Rα and Rβ. After the assignment of the anomeric signal was determined, through 1H-1H COSY, HSQC, HMBC (**Figure 2F**), and NOESY (**Figure 2G**), combined with the 1H-NMR and 13C-NMR spectra (**Table 3**), as well as the monosaccharide composition and methylation analysis results, the similar sugar residues substituted in the related literature were compared. Chemical shift data (13, 16, 17), the 1H and 13C chemical shift signals of the main types of sugar residues in the polysaccharide samples were assigned, and the results are shown in **Table 4**.

**Table 3:** Assignment of chemical shifts of 1H and 13C of each sugar residue in GPS-1.

sugar residue		chemical shift(ppm)							
			1	2	3	4	5/5a	6/5b	CH_3_- of OMe-
**A**	→4)-α-D-Gal*p*A-(1→	H	4.96	3.62	3.87	4.29	4.64		
		C	100.43	69.66	70.21	79.26	72.83	176.94	
**B**	→4)-α-D-Gal*p*A-6-OMe-(1→	H	4.80	3.62	3.85	4.44	5.02		3.67
		C	101.55	69.66	70.09	80.24	72.12	172.20	54.40
**A_1,5_**	→5)-α-L-Ara*f*-(1→	H	4.95	4.00	3.82	3.95	3.67	3.76	
		C	108.90	82.71	78.05	85.57	68.17		
**A_t_**	α-L-Ara*f*-(1→	H	5.04	4.00	3.88	4.09	3.59	3.71	
		C	108.47	82.71	78.32	83.93	62.68		
R_α_	→4)-α-D-Gal*p*A	H	5.18	3.70	3.86	4.26	–		
		C	93.69	69.38	70.09	78.87	–	176.49	
R_β_	→4)-β-D-Gal*p*A	H	4.47	3.36	3.60	4.23	3.93		
		C	97.65	72.83	71.73	78.99	75.69	176.49	

**Table 4:** Molecular weight results for GPS-1.

RT(min)	lgM_p_	lgM_w_	lgM_n_	M_p_	M_w_	M_n_	M_w_/M_n_
40.567	4.371	4.422	4.255	23505	26440	17980	1.47

The connection sequence of each sugar residue can be further inferred by HMBC remote correlation spectrum and NOESY spectrum. As shown in the HMBC correlation spectrum (**Figure 2F**) and NOESY spectrum (**Figure 2G**) of the GPS-1, the following coupling signals can be found from the Figure : (1): There is a correlation signal peak (A H-1/A C-4) between H-1 (δ 4.96 ppm) of sugar residue A in HMBC spectrum and C-4 (δ 79.26 ppm) of sugar residue A itself. In the NOESY spectrum, there is a cross peak (A H-1/A H-4) between H-1 (δ 4.96 ppm) of sugar residue An and H-4 (δ 4.29 ppm) of sugar residue An itself. These results indicate that the connection mode of →4)-α-D-GalpA-(1→4)-α-D-GalpA-(1→ exists in GPS-1 (2). There is a cross peak (A H-1/B H-4) between H-1 (δ 4.96 ppm) of sugar residue A and H-4 (δ 4.44ppm) of sugar residue B in NOESY spectrum, which indicates that there is a connection mode of →4)-α-D-GalpA-(1→4)-α-D-GalpA-6-OMe-(1→ (3) There is a cross peak (B H-1/A H-4) between H-1 (δ 4.80ppm) of sugar residue B and H-4 (δ 4.29ppm) of sugar residue An in NOESY spectrum, which indicates that there is a →4)-α-D-GalpA-6-OMe-(1→4)-α-D-GalpA-(1→connection in GPS-1 (4). There is a cross peak (At H-1/A1,5 H-4) between the H-1 (δ 5.04ppm) of the sugar residue At and the H-5a (δ 3.67ppm) of the sugar residue A1,5 in the NOESY spectrum, indicating that there is a α-Lmurf- (1–5)-α-LmurAraf-(1 “) connection in GPS-1.

According to the results of monosaccharide composition, the polysaccharide sample is mainly GalA, and the proportion of GalA is 74.39%. Combined with the results of methylation and nuclear magnetic resonance (18, 19), there are a large number of 1,4-GalpA sugar residues and a small amount of methyl esterified galacturonic acid, which accords with the domain characteristics of homogalactan (HG) pectin (20–22). It is speculated that the main chain of HG pectin is composed of →4)-α-GalpA-(1→. The results of methylation analysis and NMR (23) showed that there were arabinose residues in the polysaccharide samples, indicating that there were a few branched chains in the main chain HG-type pectin, and there were →2,4)-α-D-GalpA-(1→ sugar residues in the methylation results. Due to the low content, the NMR signal was not clear, and the molar ratio of sugar residues →4)-α-GalpA-(1→and” →2,4)-α-D-GalpA-(1→ was 61.675:1.335.

**Figure 3:**
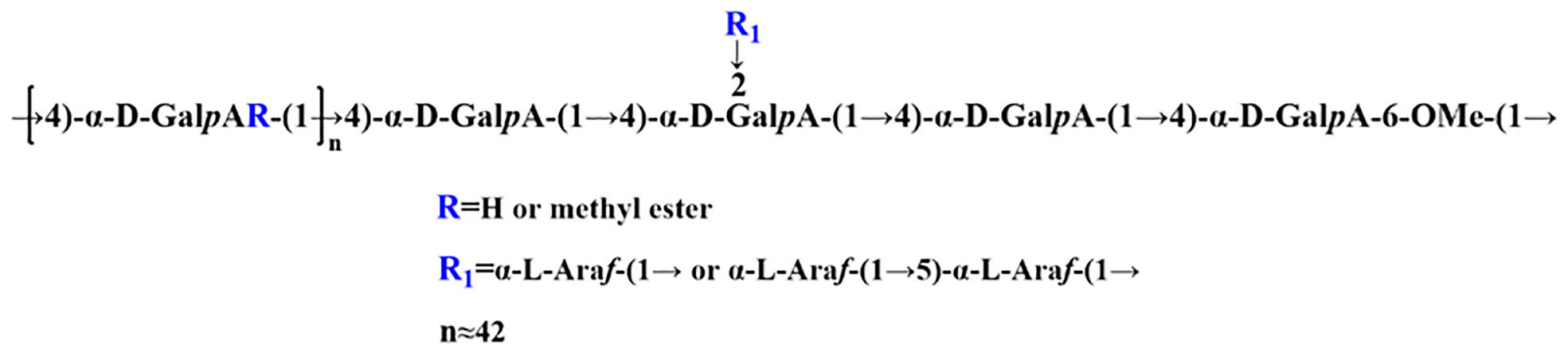
The structural model of GPS-1.

Based on the analysis of monosaccharide composition, methylation results, and one-dimensional and two-dimensional NMR (24, 25), the preliminary structure of GPS-1 polysaccharides is a homogalacturonan (HG) – type with →4)-α-D-GalpA-(1→ as the main chain. Pectin, galacturonic acid has a small amount of methyl esterification, contains a small amount of O-2 and O-3 acetylated substitutions, linked by →2,4)-α-D-GalpA-(1→ at the O-2 position Side chain, the side chain contains α-L-Araf-(1→ and →5)-α-L-Araf-(1→, and contains →4)-α-D-GalpA and →4)-β-D Two reducing end groups of -GalpA, the possible structural models of which are shown in **Figure 3**.

### 3.3 *In vivo* immune-enhancing effect of GPS-1

#### 3.3.1 GPS-1 increases serum cytokine secretion in mice

**Figure 4:**
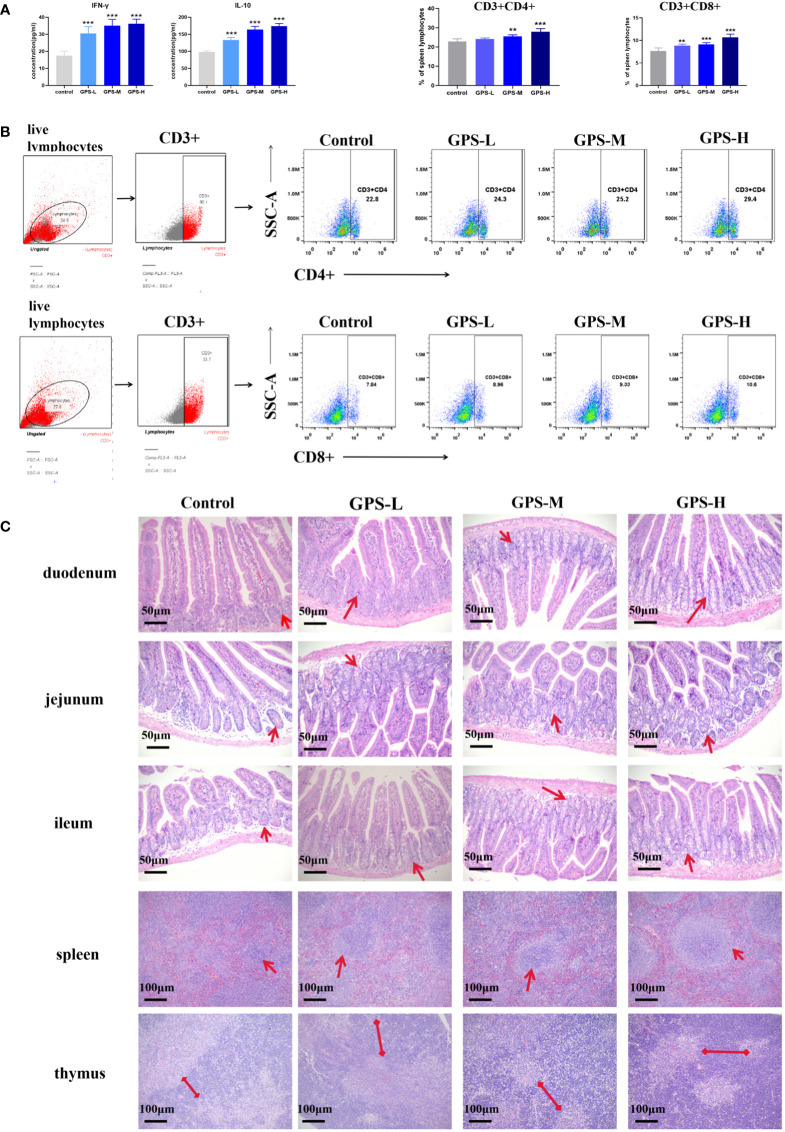
**(A)** Expression levels of IFN-γ and IL-10 in serum of mice in each group after 14 days of administration (n = 5). **(B)** Histological analysis diagram of each small intestinal segment (200×),. and thymus and spleen (100×) at 14 days after drug administration. **(C)** Expression of CD3+CD4+ and CD3+CD8+ T cells in mouse spleen lymphocytes 14 days after administration (n = 5) and representative flow scatter plots of CD3+CD4+ and CD3+CD8+ T lymphocytes.Compared with blank group (Control): **p<0.01, ***p<0.001.

As shown in **Figure 4A**, after oral administration of GPS-1 for 14 days, compared with the control group, different concentrations of GPS-1 could significantly increase the levels of IFN-γ and IL-10 in the serum of mice in a dose-dependent manner.

#### 3.3.2 GPS-1 improves the histological manifestations of small intestinal segments and immune organs in mice

As shown in **Figure 4B**, the intestines of mice in all groups were in a normal state, and the mouse intestinal villi were structurally intact and arranged in an orderly manner, without congestion or hemorrhage. Compared with the blank group (Control), the duodenal villi in each GPS-1 group were more abundant (indicated by red arrows), and the length was slightly increased; the jejunum showed an increase in the number of intestinal villi and a large increase in the number of crypts. (indicated by red arrows); histological analysis of the ileum also showed an increase in the number of crypts and intestinal villi (indicated by red arrows). Overall performance the high concentration of the GPS-1 (GPS-H) group can significantly improve the histological manifestations of the small intestine in mice.

As shown in **Figure 4B**, the HE sections of the spleen and thymus of the mice in each group were normal, without inflammatory cell infiltration, edema, congestion, and other phenomena. Spleen histology showed that compared with the blank group (Control), the germinal centers (indicated by red arrows) of the spleen in the GPS-M and GPS-H groups were more obvious, increased in number, and darker in color. The thymus results showed that the thymic cortex (indicated by the red arrow) was thickened and dark-stained in the GPS-M and GPS-H groups compared with the blank group (Control).

#### 3.3.3 GPS-1 increased the proportion of CD3 + CD4 + and CD3 + CD8 + T lymphocytes in the mouse spleen

As shown in **Figure 4C**, three concentrations of GPS-1, low, medium, and high, were effective and significantly increased the ratio of CD3+CD4+ T cells and CD3+CD8+ T cells in the spleen of mice. The proportion of CD3+CD4+ T cells increased from4.09% in the blank control group to 5.13% in the GPS-H group, an increased rate of 25.43%; the proportion of CD3+CD8+ T cells increased from 2.76% in the blank control group to 3.92%in the GPS-H group, the increase rate is 42.03%.

### 3.4 The immune-enhancing effect of GPS-1 on DCs *in vitro*

#### 3.4.1 The maximum safe concentration of GPS-1 on mouse DCs

**Figure 5:**
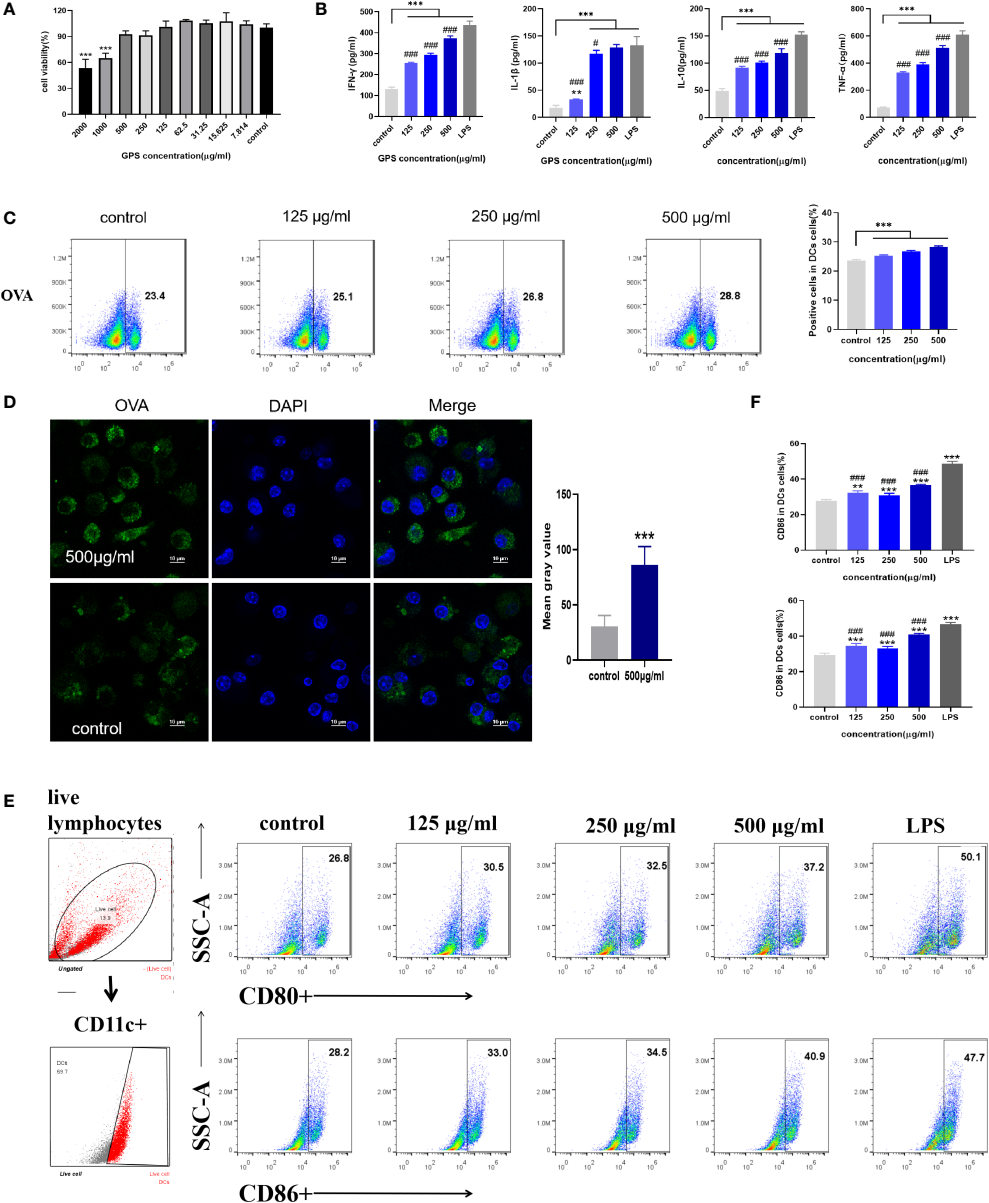
**(A)** The effect of GPS-1 on the activity of mouse DCs (n=6). **(B)** The effect of GPS-1 on cytokine secretion by DCs (n=4). **(C)** Flow cytometry and scatter diagram of GPS-1 enhancing the phagocytosis of DCs. **(D)** Confocal image of GPS-1 enhancing phagocytosis of DCs and statistics of fluorescence intensity. **(E)** GPS-1 increases the expression of CD80 and CD86 on the surface of DCs and the scatter plot.**(F)** GPS-1 increases the expression of CD80 and CD86 on DCs surface flow statistics. Compared with blank group (Control): *p<0.05, **p<0.01, ***p<0.001; compared with positive control group (LPS): #P<0.05, ##P<0.01, ###P<0.001.

The results of the determination of the maximum safe concentration of GPS-1 on mouse DCs are shown in **Figure 5A**. When the concentration of GPS-1 was in the range of 500 μg/mL~7.814 μg/mL, there was no significant difference between the cell viability of each group and the blank group (P>0.05). When the concentration of GPS-1 was greater than or equal to 1000 μg/mL, GPS-1 showed certain toxicity and significantly reduced the survival rate of DCs. This shows that GPS-1 has high safety, so we selected three concentrations under the highest safe concentration of 500μg/mL as the test concentration in the follow-up experiments.

#### 3.4.2 GPS-1 increases the secretion levels of IFN-γ, TNF-α, IL-1β, and IL-10 in mouse DCs

The expression levels of IFN-γ, TNF-α, IL-1β, and IL-10 in the cell supernatant were measured by the ELISA kit (**Figure 5B**), and the results showed that different concentrations of GPS-1 could dose-dependently increase the IFN-γ, TNF-α, IL-1β, and IL-10 in the cell supernatant, content. However, compared with the positive control group (LPS), there were also significant differences, indicating that GPS-1 could activate the activation of DCs to a certain extent.

#### 3.4.3 The effect of GPS-1 on the phagocytosis of DCs

This experiment shows the effect of GPS-1 on the phagocytic ability of DCs within the same 8 hours of culture by flow cytometry (**Figure 5C**) and laser confocal image (**Figure 5D**). The FITC-OVA fluorescence intensity of cells in each GPS-1 treatment group (500 μg/mL, 250 μg/mL, and 125 μg/mL) and the blank group (Control) were counted, and the effect of GPS-1 on the phagocytosis of DCs was calculated by flow cytometry, the results showed that 500 μg/mL, 250 μg/mL and 125 μg/mL GPS-1 could significantly promote the phagocytosis of OVA antigens by DCs. 500 μg/mL GPS-1 was selected, and the phagocytosis of OVA was observed under a confocal microscope. Compared with the blank group (Control), the OVA fluorescence intensity (green) of the 500 μg/mL GPS-1 group was significantly higher than that of the blank group.

#### 3.4.4 The effect of GPS-1 on the antigen-presenting ability of mouse DCs

As shown in **Figures 5E, F**, compared with the blank group (Control), the expression levels of co-stimulatory factors CD80 and CD86 on the surface of DCs were significantly increased after GPS-1 induction (P < 0.01). Therefore, the results indicate that GPS-1 can significantly promote the maturation and activation of DCs and the improvement of antigen presentation ability, and the positive control group (LPS) has a stronger stimulating effect than the GPS-1 group (P<0.05).

### 3.5 Study on the mechanism of GPS-1 regulating DCs activity pathway

#### 3.5.1 Repetitive assessments

**Figure 6:**
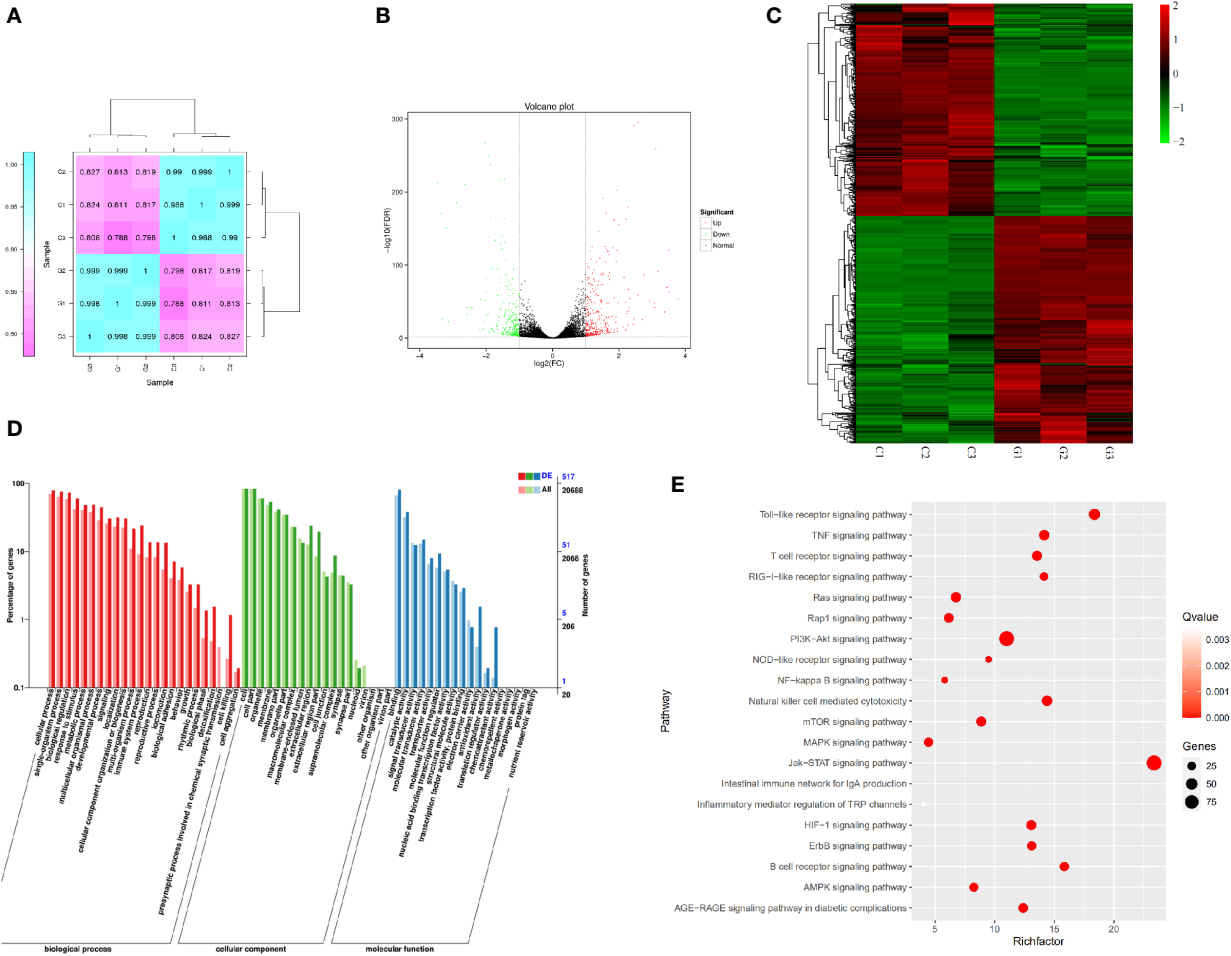
**(A)** Heat map of expression correlation of samples in different groups. **(B)** Differential gene expression volcano map. **(C)** Differential gene expression heat map. **(D)** Statistical chart of GO annotation classification of differentially expressed genes. **(E)** Bubble plot of differentially expressed genes KEGG enrichment.

Triplicate repeatability assessments (n=3) were performed between samples in each group to eliminate within-group errors as much as possible. As shown in **Figure 6A**, the gene expression differences between the GPS-1-treated cells and the blank group were obvious, and the differences between the two groups were minimal and statistically significant.

### 3.6 Screening of differential genes

As shown in **Figures 6B, C**, differentially expressed genes (DEG) analysis was performed on the transcriptome samples of the GPS-1 group and the blank control group (Control), and it was found that after GPS-1 treatment, there were 574 differences between the two groups of samples genes, of which 316 were up-regulated (red), accounting for 55.05% of all differential genes. 258 down-regulated genes (green), accounting for 44.95% of all differential genes.

### 3.7 GO classification of differentially expressed genes

As shown in **Figure 6D**, the functions of differential genes after GPS-1 treatment were mainly enriched in cellular process, single-organism process biological regulation, response to stimulus, metabolic process, and immune system process-related biological processes such as metabolism and immune regulation. (red); cell part, membrane part, organelle, and cell junction-related molecular functions such as secretion and cell junction (green); binding, catalytic activity, molecular transducer function activity, and other related cellular components such as catalysis and transformation (blue) changes.

#### 3.7.1 KEGG annotation of differentially expressed genes

KEGG annotation analysis was performed on the differential genes. The results shown in **Figure 6E** show that among immune-related pathways, the Toll-like receptors pathway, PI3K-Akt pathway, and Jak-STAT pathway are more highly enriched and more significant.

## 4 Discussion

Licorice is a traditional plant that is widely used for medicine and food. The analysis of glycyrrhiza components is also quite numerous. Among them, Licorice Polysaccharide, as a macromolecular carbohydrate, has been extensively studied in recent years due to its unique biological activity. Zhang et al. (26) extracted a new neutral polysaccharide AGP from glycyrrhiza residue with a molecular weight of 2.89 × 10 KDa, monosaccharide composition analysis indicated that AGP consisted of l-rhamnose: l-arabinose: d-xylose: d-mannose: d-glucose and d-galactose with a molar ratio of 1:2.33:2.85:0.69:3.05:1.54. AGP was composed of → 6)-β-d-Glcp-(→ backbone and the →4)-α-d-Xylp-(1→, →5)-α-l-Araf-(1→, →3) -α-l-Rhap-(1→, →6)-α-d-Galp-(1→, →3,6)-α-Manp-(1→ and →1)-β-d-Glcp as branche. Pan LC et al. (27) extracted a homogeneous polysaccharide GIBP from Glycyrrhiza Chinensis with a backbone composed of α-D-1,4-linked glucose and branch points composed of α-D-1,3,6 and α-D -1,2,3,6-linked glucose with side chains consisting of α-D-1,3 and β-D-1,6-linked galactose, β-L-1,2-linked arabinose, α- It is composed of D-1,3 and β-D-1,3, and its activity is mainly manifested in antioxidant aspects such as the scavenging ability of DPPH radicals, OH radicals, O radicals and ABTS. Mutaillifu P et al. (28) reported a polysaccharide GPN extracted from Glycyrrhiza glabra with a molecular weight of 38.7 kDa. Monosaccharide composition analysis confirmed the presence of major glucose (98.03%) and trace amounts of mannose, arabinose, and galactose, the main glycosidic bonds in GPN comprised 1,4-linked Glcp, T-linked Glcp, 1,4,6-linked Glcp, and 1,6-linked Glcp. It can be seen from the above that the structure of Glycyrrhiza polysaccharide is complex and diverse, such as the degree of polymerization, the composition and connection sequence of sugar units, branched-chain structure and higher-order structure, etc., which directly affect its biological activity. A large number of studies have shown that Glycyrrhiza polysaccharides have strong pharmacological activities, such as antioxidant (29), immune regulation (30), anti-tumor (31), apoptosis (32), anti-microbial (33), anti-inflammatory (34), and intestinal flora regulation (35). This may be the different biological activities caused by different polysaccharide structures. In this study, the immunomodulatory activity of polyrhamnogalacturonic acid-type pectin-type polysaccharide GPS-1 was evaluated. First of all, in terms of immune regulation, GPS-1 showed an effective immune-enhancing function. And the results showed (**Figure 3**) that GPS-1 could effectively increase the secretion of mouse cytokines IFN-γ and IL-10 which are secreted by Th1-type cells and Th2-type cells, respectively. Correspondingly, the proportion of CD3+CD4+ (Th2) cells and CD3+CD8+ (Th2) cells in spleen lymphocytes also showed a dose-dependent increase. Intuitively, GPS-1 improve the histological manifestations of intestinal and immune organs. (**Figures 4A, D**). *In vitro* experiments, GPS-1 can effectively promote DCs to secrete IFN-γ, TNF-α, IL-1β, and IL-10, these cytokines can make quiescent lymphocytes differentiate into functional lymphocytes. At the same time, it promoted the increase of the expression levels of CD80 and CD86 on the surface of DCs. The expression levels of CD80 and CD86 reflected the maturity of DCs, and mature DCs could play the function of antigen presentation more effectively. Antigen uptake assay showed that GPS-1 could help DCs phagocytose more antigens. To sum up, GPS-1 can help DCs to better exert their immune ability, promote lymphocyte differentiation, and help the body to obtain a higher immune level. Also in this experiment, polysaccharide GPS-1 also showed the immunomodulatory activity and the effect of improving intestinal immunity as reported in the above literature. But its structure is reported for the first time, GPS-1 is a polygalacturonic acid-type pectin, namely HG-type pectin. (**Figure 3**). Polygalacturonic acid HG is the most abundant pectin polysaccharide in plants. It is reported that some scholars have pointed out that the intake of pectin polysaccharide can be a therapeutic strategy for managing intestinal inflammation. Among them, anti-inflammatory cytokines and intestinal barrier function tend to be regulated by galactan-rich pectic polysaccharides (4). Since GPS-1 is a pectin polysaccharide rich in galacturonic acid, it has a positive effect on improving intestinal immunity, which is consistent with the previous study in our laboratory that GPS-1 can improve the intestinal immunity of laying hens. In laying hens, the addition of glycyrrhiza polysaccharide in the feed can regulate the abundance of intestinal flora and promote the increase of beneficial bacteria (4). It also suggested the effect of this type of polysaccharide on the intestinal barrier and immune function. Provide a scientific basis for the relationship between polysaccharide configuration and biological activity.

Preliminary studies have shown that Glycyrrhiza polysaccharides can affect signal transduction between cells, especially immune cells (6, 36). Glycyrrhiza polysaccharide can significantly promote the maturation and cytokine secretion of human monocyte-derived dendritic cells and mouse bone marrow-derived dendritic cells, which may be through toll-like receptor 4 (TLR4), downstream p38 signaling pathway, amino-terminal Kinase (JNK) and nuclear factor κb (NF-κB) signaling (8). Focusing on the pathways related to the regulation of immune cell function by polysaccharides, it can be found that polysaccharides such as Ganoderma lucidum polysaccharides and Lycium barbarum polysaccharides all further activate the intracellular MAPK and NF-κB signaling pathways by affecting the cellular TLR-2 and TLR-4 pathways (37, 38). In this study, we explored the pathway of GPS-1 affecting DCs through transcriptomics, analyzed the differences in the transcripts of DCs in GPS-1 intervention and DCs in the normal state (**Figures 6A–C**), and found the differential gene expression of cells in this state and the normal group to speculate on the pathways and mechanisms of the differences. Finally, the results we obtained through the analysis of transcriptome data GO (**Figure 6D**) and KEGG (**Figure 6E**) is consistent with the above literature reports and activate cellular immune function through the TLR pathway. At the same time, we also found that GPS-1 affects the downstream TLR-2 and TLR-4 pathways, ie PI3K-AKT signaling pathway. And with this pathway as the core, it also acts on various downstream pathways such as MAPK, NF-κB, NOD, mTOR, and AMPK to regulate the activation and maturation of DC cells in the nucleus, and various cytokines secreted in turn act on the cells. JAK-STAT signaling pathway on DCs cells. In this way, the multi-target effect of GPS-1 on DCs can be realized, and various cellular functions such as growth, differentiation, apoptosis, and immune regulation of DCs can be comprehensively adjusted.

## 5 Conclusion

A novel HG-type pectin glycopolysaccharide, glycyrrhiza polysaccharides GPS-1, is composed of fucose, rhamnose, arabinose, galactose, glucose, xylose, galacturonic acid, and mannuronic acid in the percentages 0.92%, 10.12%, 6.36%, 4.85%, 1.34%, 0.51%, 74.39%, and 1.52%. The main chain of GPS-1 The structure is composed of →4)-α-D-GalpA-(1→.

GPS-1 was able to show good immunomodulatory activity in both *in vitro* and *in vivo* experiments. In particular, it can effectively promote the phagocytosis and antigen-presenting ability of DCs in regulating the immune activity of dendritic cells *in vitro*. At the same time, the transcriptomic analysis revealed that GPS-1 influenced the TLR-2 and TLR-4 pathways, and further regulated the viability of DCs through the PI3K- AKT signaling pathway and various downstream signaling pathways.

## Data availability statement

The datasets presented in this study can be found in online repositories. The names of the repository/repositories and accession number(s) can be found below: https://www.ncbi.nlm.nih.gov/, PRJNA864785.

## Ethics statement

The animal study was reviewed and approved by Institutional Animal Care and Use Committee Institutional Animal Care and Use Committee.

## Author contributions

YuW and HZ, data curation and writing-original draft preparation. KW, writing-original draft preparation, methodology, and visualization. TZ, methodology and editing. YC, writing-reviewing and methodology. AN, SP, BZ, XY, and YL, writing-reviewing. XW, PH, and LW, data validation. XC, data curation. CL and HL visualization. JL, methodology. YiW, conceptualization, funding acquisition, project administration, and supervision. All authors contributed to the article and approved the submitted version.

## Funding

This research was financially supported by the National Natural Science Foundation of China (NSFC, Grant No. 31872514 and 32172900), the Open Project Program of Beijing Key Laboratory of 490 Traditional Chinese Veterinary Medicine at Beijing University of Agriculture (No. kftcvm202101), Yunnan Provincial Science and Technology Department-Applied Basic Research Joint Special Funds of Yunnan University of Chinese Medicine [2018FF001 (-020), 2019FF002(-012)], Guangxi Science and Technology Project (GuiKe ZY20198018, GuiKe AA18242040) and a project funded by the Priority Academic Program Development of Jiangsu Higher Education Institutions (PAPD). We appreciate the assistance from our distinguished colleagues in the Institute of Traditional Chinese Veterinary Medicine of Nanjing Agricultural University and the technological managers in Nanjing Super Biotech Co., Ltd.

## Conflict of interest

The authors declare that the research was conducted in the absence of any commercial or financial relationships that could be construed as a potential conflict of interest.

## Publisher’s note

All claims expressed in this article are solely those of the authors and do not necessarily represent those of their affiliated organizations, or those of the publisher, the editors and the reviewers. Any product that may be evaluated in this article, or claim that may be made by its manufacturer, is not guaranteed or endorsed by the publisher.
